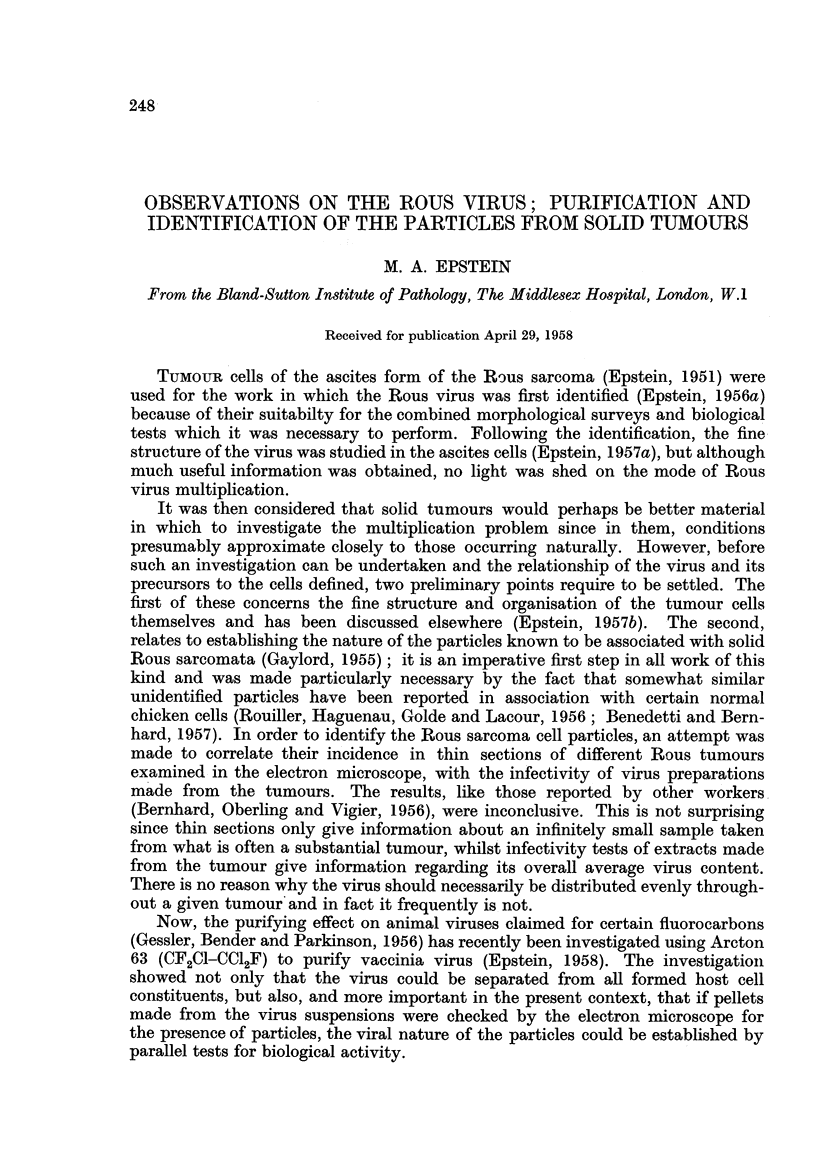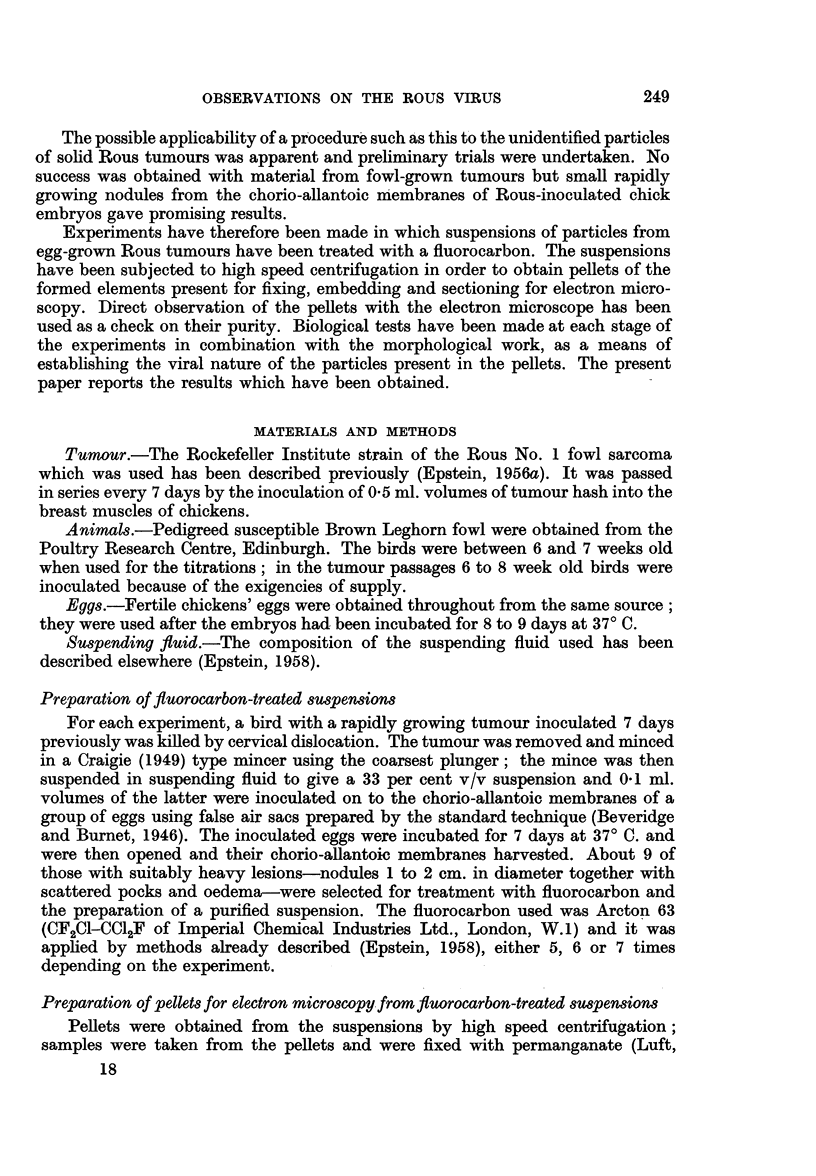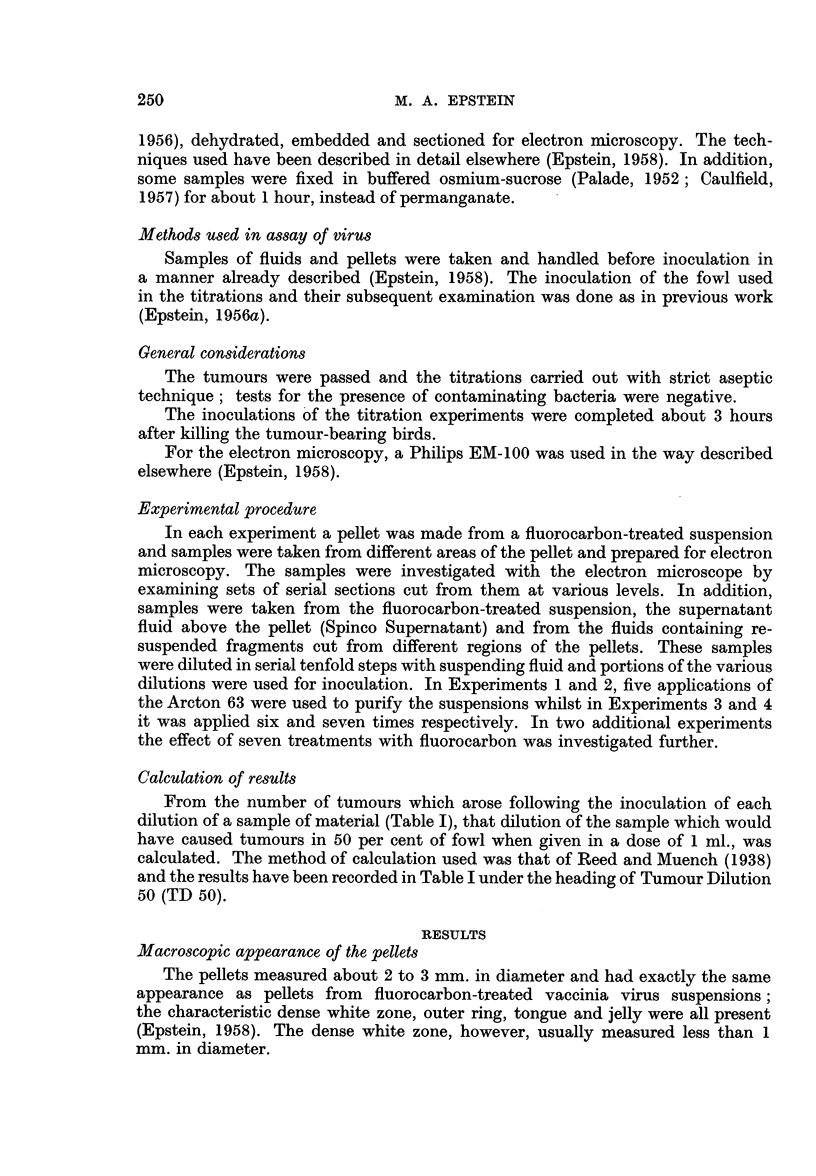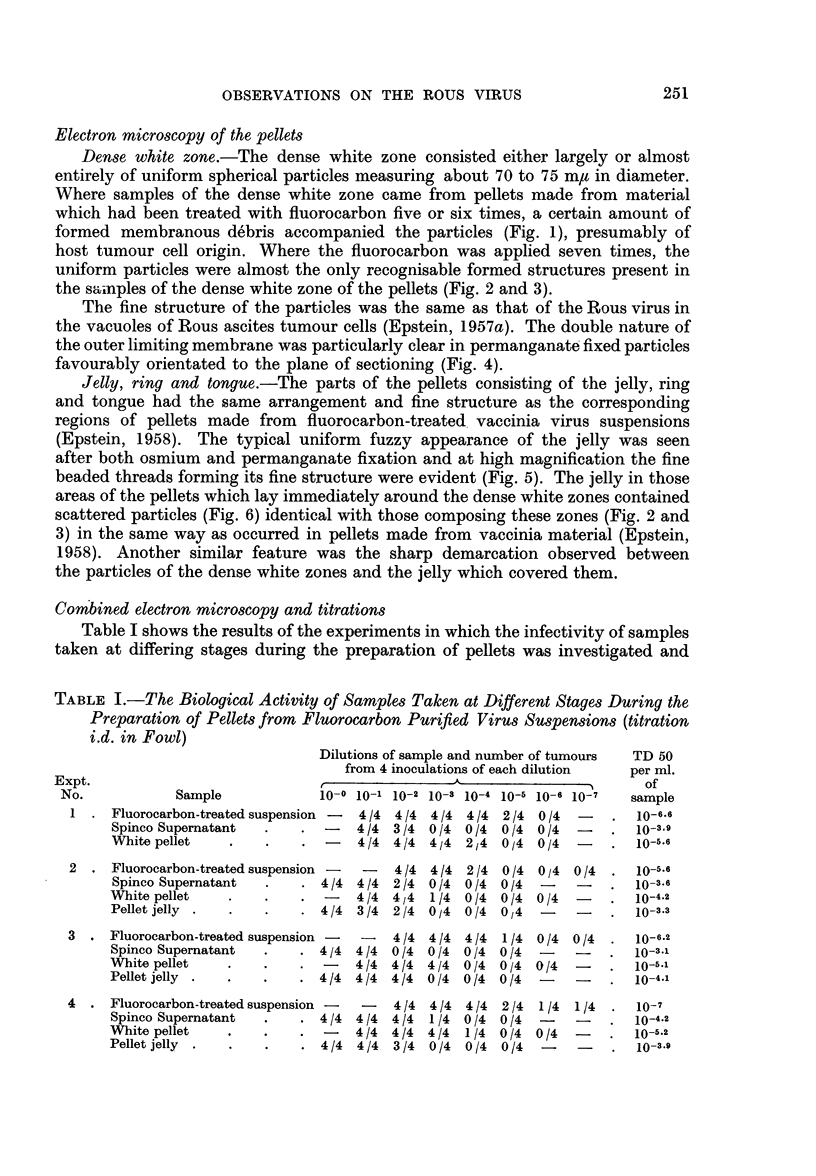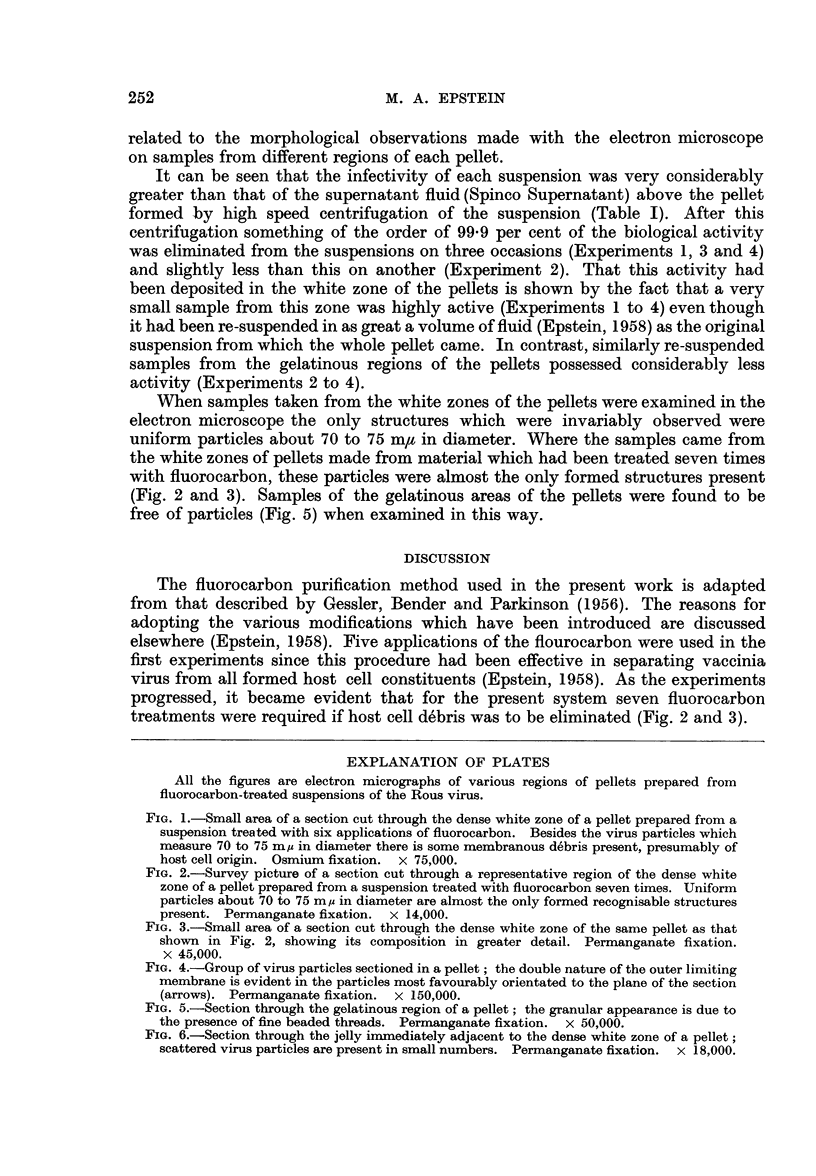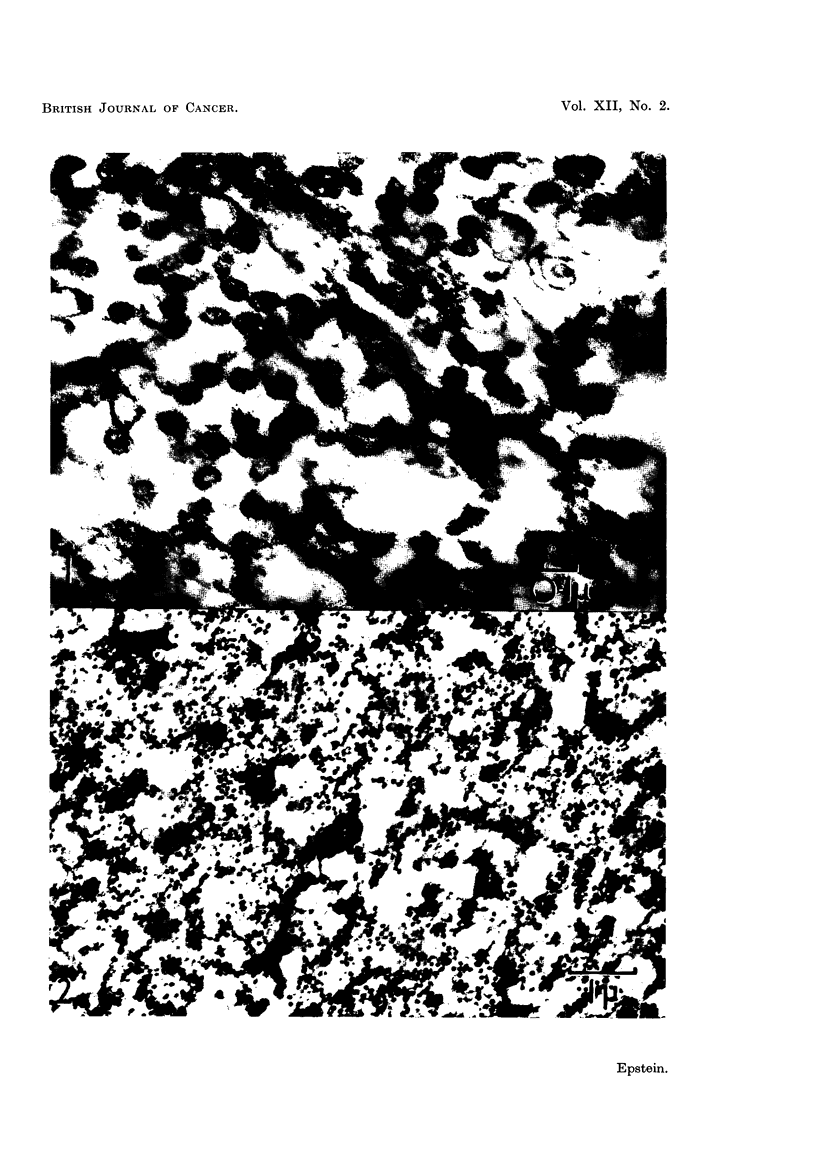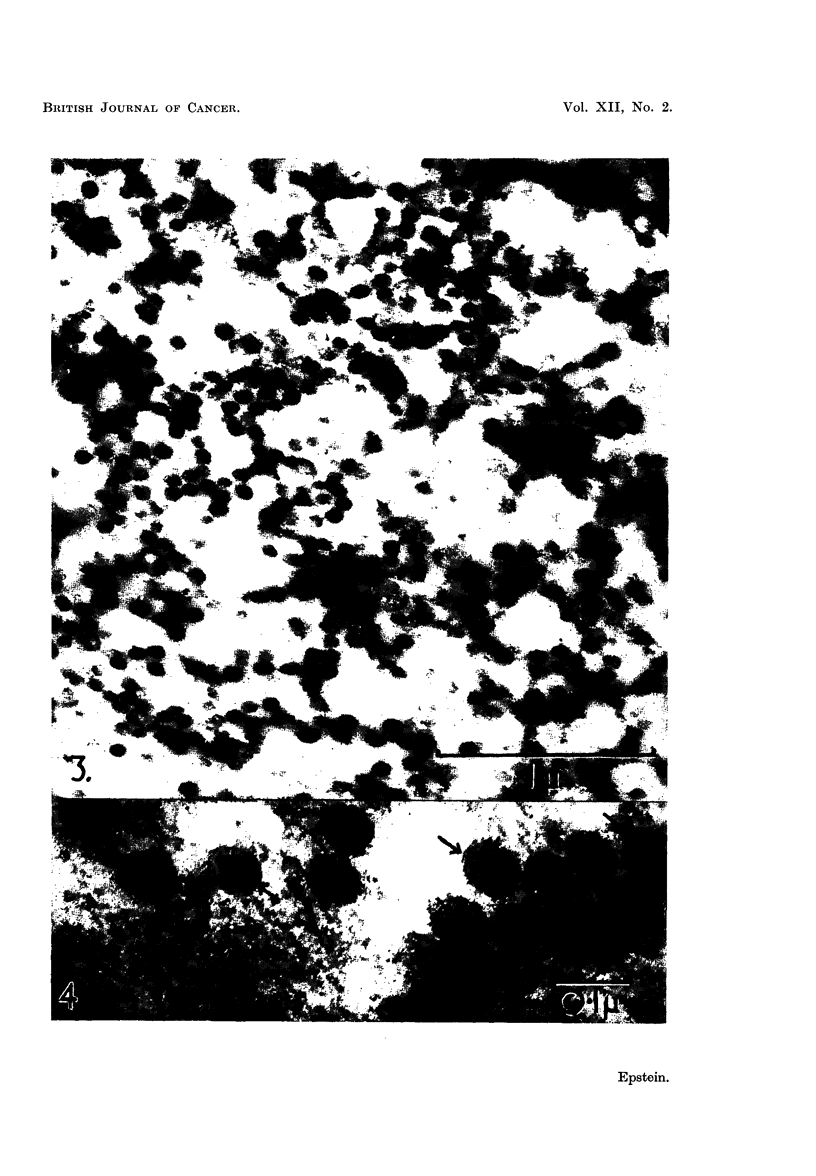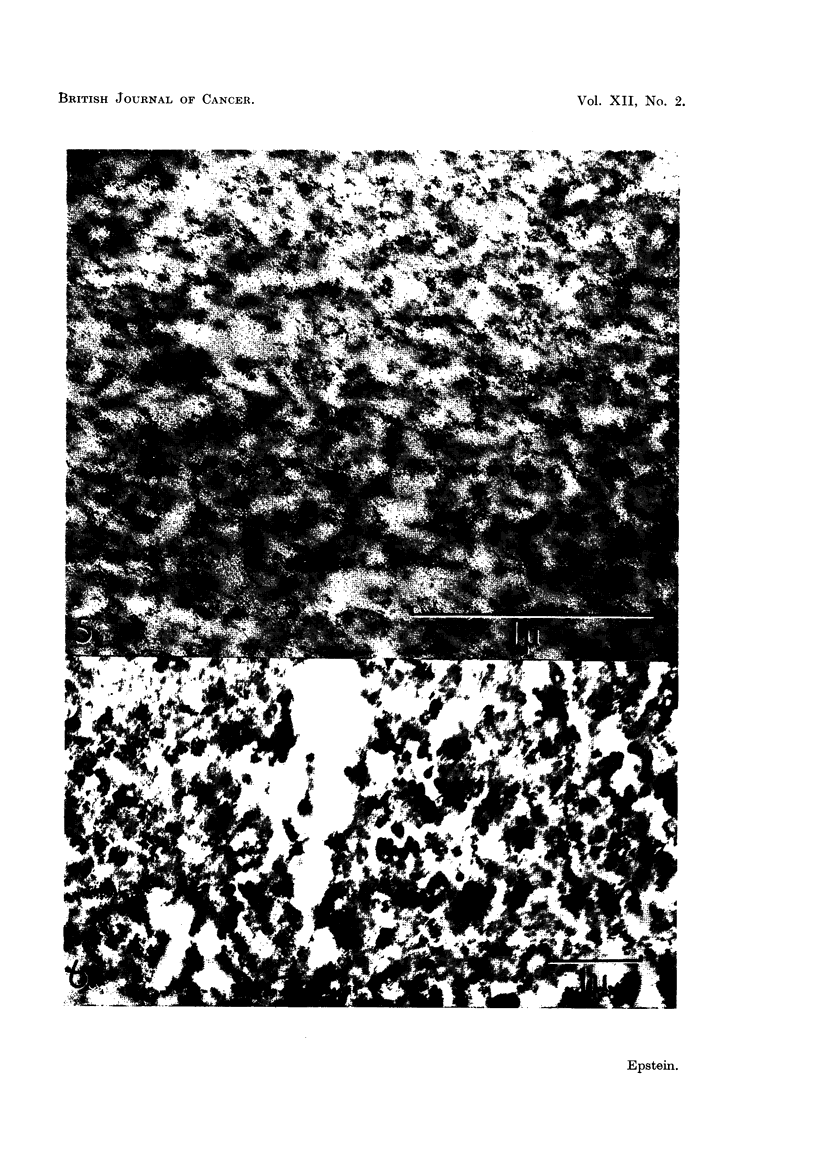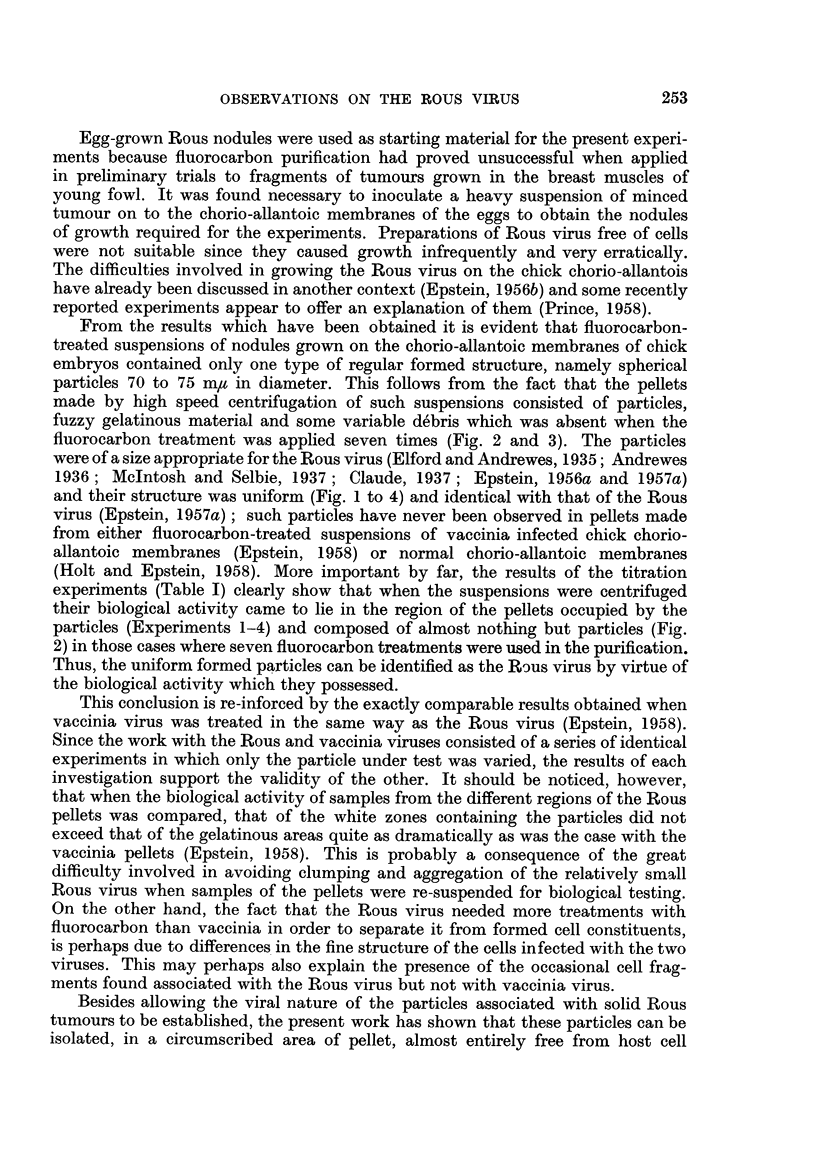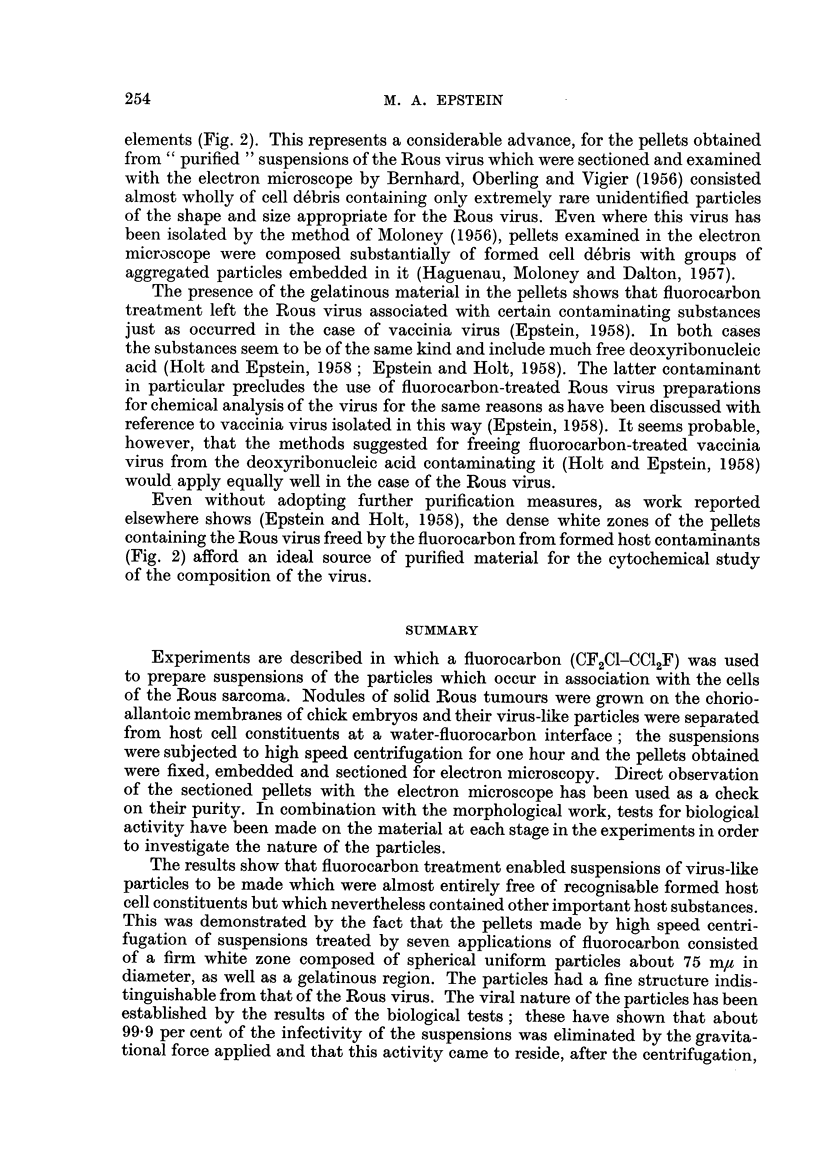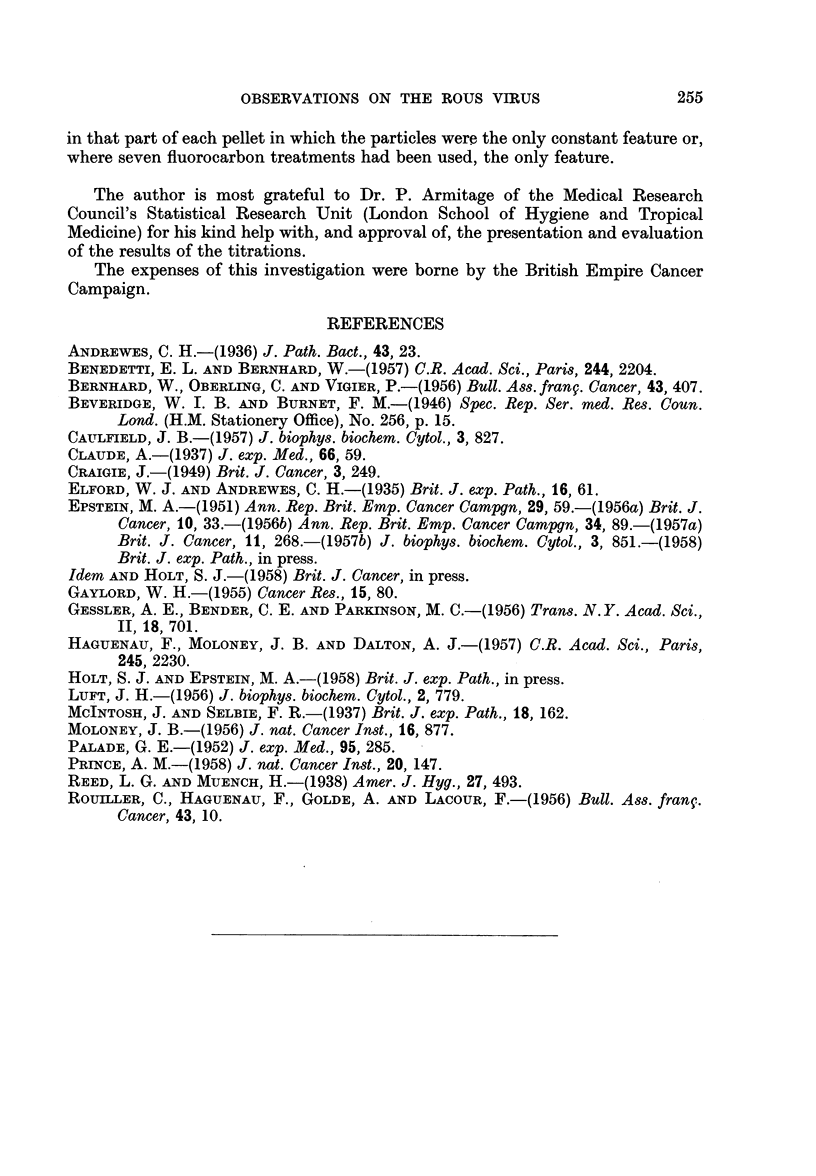# Observations on the Rous Virus; Purification and Identification of the Particles from Solid Tumours

**DOI:** 10.1038/bjc.1958.30

**Published:** 1958-06

**Authors:** M. A. Epstein

## Abstract

**Images:**


### OBSERVATIONS ON THE ROUS VIRUS; PURIFICATION AND IDENTIFICATION OF THE PARTICLES FROM SOLID TUMOURS

M. A. EPSTEIN

*From the Bland-Sutton Institute of Pathology, The Middlesex Hospital, London, W.1*

Received for publication April 29, 1958

TUMOUR cells of the ascites form of the Rous sarcoma (Epstein, 1951) were used for the work in which the Rous virus was first identified (Epstein, 1956*a*) because of their suitabilty for the combined morphological surveys and biological tests which it was necessary to perform. Following the identification, the fine structure of the virus was studied in the ascites cells (Epstein, 1957*a*), but although much useful information was obtained, no light was shed on the mode of Rous virus multiplication.

It was then considered that solid tumours would perhaps be better material in which to investigate the multiplication problem since in them, conditions presumably approximate closely to those occurring naturally. However, before such an investigation can be undertaken and the relationship of the virus and its precursors to the cells defined, two preliminary points require to be settled. The first of these concerns the fine structure and organisation of the tumour cells themselves and has been discussed elsewhere (Epstein, 1957*b*). The second, relates to establishing the nature of the particles known to be associated with solid Rous sarcomata (Gaylord, 1955); it is an imperative first step in all work of this kind and was made particularly necessary by the fact that somewhat similar unidentified particles have been reported in association with certain normal chicken cells (Rouiller, Haguenau, Golde and Lacour, 1956; Benedetti and Bernhard, 1957). In order to identify the Rous sarcoma cell particles, an attempt was made to correlate their incidence in thin sections of different Rous tumours examined in the electron microscope, with the infectivity of virus preparations made from the tumours. The results, like those reported by other workers (Bernhard, Oberling and Vigier, 1956), were inconclusive. This is not surprising since thin sections only give information about an infinitely small sample taken from what is often a substantial tumour, whilst infectivity tests of extracts made from the tumour give information regarding its overall average virus content. There is no reason why the virus should necessarily be distributed evenly throughout a given tumour and in fact it frequently is not.

Now, the purifying effect on animal viruses claimed for certain fluorocarbons (Gessler, Bender and Parkinson, 1956) has recently been investigated using Arcton 63 ($CF_2Cl$–$CCl_2F$) to purify vaccinia virus (Epstein, 1958). The investigation showed not only that the virus could be separated from all formed host cell constituents, but also, and more important in the present context, that if pellets made from the virus suspensions were checked by the electron microscope for the presence of particles, the viral nature of the particles could be established by parallel tests for biological activity.

The possible applicability of a procedure such as this to the unidentified particles of solid Rous tumours was apparent and preliminary trials were undertaken. No success was obtained with material from fowl-grown tumours but small rapidly growing nodules from the chorio-allantoic membranes of Rous-inoculated chick embryos gave promising results.

Experiments have therefore been made in which suspensions of particles from egg-grown Rous tumours have been treated with a fluorocarbon. The suspensions have been subjected to high speed centrifugation in order to obtain pellets of the formed elements present for fixing, embedding and sectioning for electron microscopy. Direct observation of the pellets with the electron microscope has been used as a check on their purity. Biological tests have been made at each stage of the experiments in combination with the morphological work, as a means of establishing the viral nature of the particles present in the pellets. The present paper reports the results which have been obtained.

### MATERIALS AND METHODS

*Tumour.*—The Rockefeller Institute strain of the Rous No. 1 fowl sarcoma which was used has been described previously (Epstein, 1956*a*). It was passed in series every 7 days by the inoculation of 0·5 ml. volumes of tumour hash into the breast muscles of chickens.

*Animals.*—Pedigreed susceptible Brown Leghorn fowl were obtained from the Poultry Research Centre, Edinburgh. The birds were between 6 and 7 weeks old when used for the titrations ; in the tumour passages 6 to 8 week old birds were inoculated because of the exigencies of supply.

*Eggs.*—Fertile chickens' eggs were obtained throughout from the same source ; they were used after the embryos had been incubated for 8 to 9 days at 37° C.

*Suspending fluid.*—The composition of the suspending fluid used has been described elsewhere (Epstein, 1958).

### *Preparation of fluorocarbon-treated suspensions*

For each experiment, a bird with a rapidly growing tumour inoculated 7 days previously was killed by cervical dislocation. The tumour was removed and minced in a Craigie (1949) type mincer using the coarsest plunger ; the mince was then suspended in suspending fluid to give a 33 per cent v/v suspension and 0·1 ml. volumes of the latter were inoculated on to the chorio-allantoic membranes of a group of eggs using false air sacs prepared by the standard technique (Beveridge and Burnet, 1946). The inoculated eggs were incubated for 7 days at 37° C. and were then opened and their chorio-allantoic membranes harvested. About 9 of those with suitably heavy lesions—nodules 1 to 2 cm. in diameter together with scattered pocks and oedema—were selected for treatment with fluorocarbon and the preparation of a purified suspension. The fluorocarbon used was Arcton 63 ($CF_2Cl–CCl_2F$ of Imperial Chemical Industries Ltd., London, W.1) and it was applied by methods already described (Epstein, 1958), either 5, 6 or 7 times depending on the experiment.

### *Preparation of pellets for electron microscopy from fluorocarbon-treated suspensions*

Pellets were obtained from the suspensions by high speed centrifugation ; samples were taken from the pellets and were fixed with permanganate (Luft,

1956), dehydrated, embedded and sectioned for electron microscopy. The techniques used have been described in detail elsewhere (Epstein, 1958). In addition, some samples were fixed in buffered osmium-sucrose (Palade, 1952 ; Caulfield, 1957) for about 1 hour, instead of permanganate.

*Methods used in assay of virus*

Samples of fluids and pellets were taken and handled before inoculation in a manner already described (Epstein, 1958). The inoculation of the fowl used in the titrations and their subsequent examination was done as in previous work (Epstein, 1956*a*).

*General considerations*

The tumours were passed and the titrations carried out with strict aseptic technique ; tests for the presence of contaminating bacteria were negative.

The inoculations of the titration experiments were completed about 3 hours after killing the tumour-bearing birds.

For the electron microscopy, a Philips EM-100 was used in the way described elsewhere (Epstein, 1958).

*Experimental procedure*

In each experiment a pellet was made from a fluorocarbon-treated suspension and samples were taken from different areas of the pellet and prepared for electron microscopy. The samples were investigated with the electron microscope by examining sets of serial sections cut from them at various levels. In addition, samples were taken from the fluorocarbon-treated suspension, the supernatant fluid above the pellet (Spinco Supernatant) and from the fluids containing resuspended fragments cut from different regions of the pellets. These samples were diluted in serial tenfold steps with suspending fluid and portions of the various dilutions were used for inoculation. In Experiments 1 and 2, five applications of the Arcton 63 were used to purify the suspensions whilst in Experiments 3 and 4 it was applied six and seven times respectively. In two additional experiments the effect of seven treatments with fluorocarbon was investigated further.

*Calculation of results*

From the number of tumours which arose following the inoculation of each dilution of a sample of material (Table I), that dilution of the sample which would have caused tumours in 50 per cent of fowl when given in a dose of 1 ml., was calculated. The method of calculation used was that of Reed and Muench (1938) and the results have been recorded in Table I under the heading of Tumour Dilution 50 (TD 50).

### RESULTS

*Macroscopic appearance of the pellets*

The pellets measured about 2 to 3 mm. in diameter and had exactly the same appearance as pellets from fluorocarbon-treated vaccinia virus suspensions ; the characteristic dense white zone, outer ring, tongue and jelly were all present (Epstein, 1958). The dense white zone, however, usually measured less than 1 mm. in diameter.

*Electron microscopy of the pellets*

*Dense white zone.*—The dense white zone consisted either largely or almost entirely of uniform spherical particles measuring about 70 to 75 m$\mu$ in diameter. Where samples of the dense white zone came from pellets made from material which had been treated with fluorocarbon five or six times, a certain amount of formed membranous débris accompanied the particles (Fig. 1), presumably of host tumour cell origin. Where the fluorocarbon was applied seven times, the uniform particles were almost the only recognisable formed structures present in the samples of the dense white zone of the pellets (Fig. 2 and 3).

The fine structure of the particles was the same as that of the Rous virus in the vacuoles of Rous ascites tumour cells (Epstein, 1957*a*). The double nature of the outer limiting membrane was particularly clear in permanganate fixed particles favourably orientated to the plane of sectioning (Fig. 4).

*Jelly, ring and tongue.*—The parts of the pellets consisting of the jelly, ring and tongue had the same arrangement and fine structure as the corresponding regions of pellets made from fluorocarbon-treated vaccinia virus suspensions (Epstein, 1958). The typical uniform fuzzy appearance of the jelly was seen after both osmium and permanganate fixation and at high magnification the fine beaded threads forming its fine structure were evident (Fig. 5). The jelly in those areas of the pellets which lay immediately around the dense white zones contained scattered particles (Fig. 6) identical with those composing these zones (Fig. 2 and 3) in the same way as occurred in pellets made from vaccinia material (Epstein, 1958). Another similar feature was the sharp demarcation observed between the particles of the dense white zones and the jelly which covered them.

*Combined electron microscopy and titrations*

Table I shows the results of the experiments in which the infectivity of samples taken at differing stages during the preparation of pellets was investigated and

TABLE I.—*The Biological Activity of Samples Taken at Different Stages During the Preparation of Pellets from Fluorocarbon Purified Virus Suspensions (titration i.d. in Fowl)*

| Expt. No. | Sample | Dilutions of sample and number of tumours from 4 inoculations of each dilution | | | | | | | | TD 50 per ml. of sample |
|---|---|---|---|---|---|---|---|---|---|---|
| | | $10^{-0}$ | $10^{-1}$ | $10^{-2}$ | $10^{-3}$ | $10^{-4}$ | $10^{-5}$ | $10^{-6}$ | $10^{-7}$ | |
| 1 | Fluorocarbon-treated suspension | — | 4/4 | 4/4 | 4/4 | 4/4 | 2/4 | 0/4 | — | $10^{-6.6}$ |
| | Spinco Supernatant | — | 4/4 | 3/4 | 0/4 | 0/4 | 0/4 | 0/4 | — | $10^{-3.9}$ |
| | White pellet | — | 4/4 | 4/4 | 4/4 | 2/4 | 0/4 | 0/4 | — | $10^{-5.6}$ |
| 2 | Fluorocarbon-treated suspension | — | — | 4/4 | 4/4 | 2/4 | 0/4 | 0/4 | 0/4 | $10^{-5.6}$ |
| | Spinco Supernatant | 4/4 | 4/4 | 2/4 | 0/4 | 0/4 | 0/4 | — | — | $10^{-3.6}$ |
| | White pellet | — | 4/4 | 4/4 | 1/4 | 0/4 | 0/4 | 0/4 | — | $10^{-4.2}$ |
| | Pellet jelly | 4/4 | 3/4 | 2/4 | 0/4 | 0/4 | 0/4 | — | — | $10^{-3.3}$ |
| 3 | Fluorocarbon-treated suspension | — | — | 4/4 | 4/4 | 4/4 | 1/4 | 0/4 | 0/4 | $10^{-6.2}$ |
| | Spinco Supernatant | 4/4 | 4/4 | 0/4 | 0/4 | 0/4 | 0/4 | — | — | $10^{-3.1}$ |
| | White pellet | — | 4/4 | 4/4 | 4/4 | 0/4 | 0/4 | 0/4 | — | $10^{-5.1}$ |
| | Pellet jelly | 4/4 | 4/4 | 4/4 | 0/4 | 0/4 | 0/4 | — | — | $10^{-4.1}$ |
| 4 | Fluorocarbon-treated suspension | — | — | 4/4 | 4/4 | 4/4 | 2/4 | 1/4 | 1/4 | $10^{-7}$ |
| | Spinco Supernatant | 4/4 | 4/4 | 4/4 | 1/4 | 0/4 | 0/4 | — | — | $10^{-4.2}$ |
| | White pellet | — | 4/4 | 4/4 | 4/4 | 1/4 | 0/4 | 0/4 | — | $10^{-5.2}$ |
| | Pellet jelly | 4/4 | 4/4 | 3/4 | 0/4 | 0/4 | 0/4 | — | — | $10^{-3.9}$ |

related to the morphological observations made with the electron microscope on samples from different regions of each pellet.

It can be seen that the infectivity of each suspension was very considerably greater than that of the supernatant fluid (Spinco Supernatant) above the pellet formed by high speed centrifugation of the suspension (Table I). After this centrifugation something of the order of 99·9 per cent of the biological activity was eliminated from the suspensions on three occasions (Experiments 1, 3 and 4) and slightly less than this on another (Experiment 2). That this activity had been deposited in the white zone of the pellets is shown by the fact that a very small sample from this zone was highly active (Experiments 1 to 4) even though it had been re-suspended in as great a volume of fluid (Epstein, 1958) as the original suspension from which the whole pellet came. In contrast, similarly re-suspended samples from the gelatinous regions of the pellets possessed considerably less activity (Experiments 2 to 4).

When samples taken from the white zones of the pellets were examined in the electron microscope the only structures which were invariably observed were uniform particles about 70 to 75 m$\mu$ in diameter. Where the samples came from the white zones of pellets made from material which had been treated seven times with fluorocarbon, these particles were almost the only formed structures present (Fig. 2 and 3). Samples of the gelatinous areas of the pellets were found to be free of particles (Fig. 5) when examined in this way.

### DISCUSSION

The fluorocarbon purification method used in the present work is adapted from that described by Gessler, Bender and Parkinson (1956). The reasons for adopting the various modifications which have been introduced are discussed elsewhere (Epstein, 1958). Five applications of the flourocarbon were used in the first experiments since this procedure had been effective in separating vaccinia virus from all formed host cell constituents (Epstein, 1958). As the experiments progressed, it became evident that for the present system seven fluorocarbon treatments were required if host cell débris was to be eliminated (Fig. 2 and 3).

---

### EXPLANATION OF PLATES

All the figures are electron micrographs of various regions of pellets prepared from fluorocarbon-treated suspensions of the Rous virus.

FIG. 1.—Small area of a section cut through the dense white zone of a pellet prepared from a suspension treated with six applications of fluorocarbon. Besides the virus particles which measure 70 to 75 m$\mu$ in diameter there is some membranous débris present, presumably of host cell origin. Osmium fixation. $\times$ 75,000.

FIG. 2.—Survey picture of a section cut through a representative region of the dense white zone of a pellet prepared from a suspension treated with fluorocarbon seven times. Uniform particles about 70 to 75 m$\mu$ in diameter are almost the only formed recognisable structures present. Permanganate fixation. $\times$ 14,000.

FIG. 3.—Small area of a section cut through the dense white zone of the same pellet as that shown in Fig. 2, showing its composition in greater detail. Permanganate fixation. $\times$ 45,000.

FIG. 4.—Group of virus particles sectioned in a pellet ; the double nature of the outer limiting membrane is evident in the particles most favourably orientated to the plane of the section (arrows). Permanganate fixation. $\times$ 150,000.

FIG. 5.—Section through the gelatinous region of a pellet ; the granular appearance is due to the presence of fine beaded threads. Permanganate fixation. $\times$ 50,000.

FIG. 6.—Section through the jelly immediately adjacent to the dense white zone of a pellet ; scattered virus particles are present in small numbers. Permanganate fixation. $\times$ 18,000.

Epstein.

Epstein.

Epstein.

Egg-grown Rous nodules were used as starting material for the present experiments because fluorocarbon purification had proved unsuccessful when applied in preliminary trials to fragments of tumours grown in the breast muscles of young fowl. It was found necessary to inoculate a heavy suspension of minced tumour on to the chorio-allantoic membranes of the eggs to obtain the nodules of growth required for the experiments. Preparations of Rous virus free of cells were not suitable since they caused growth infrequently and very erratically. The difficulties involved in growing the Rous virus on the chick chorio-allantois have already been discussed in another context (Epstein, 1956*b*) and some recently reported experiments appear to offer an explanation of them (Prince, 1958).

From the results which have been obtained it is evident that fluorocarbon-treated suspensions of nodules grown on the chorio-allantoic membranes of chick embryos contained only one type of regular formed structure, namely spherical particles 70 to 75 m$\mu$ in diameter. This follows from the fact that the pellets made by high speed centrifugation of such suspensions consisted of particles, fuzzy gelatinous material and some variable dèbris which was absent when the fluorocarbon treatment was applied seven times (Fig. 2 and 3). The particles were of a size appropriate for the Rous virus (Elford and Andrewes, 1935; Andrewes 1936; McIntosh and Selbie, 1937; Claude, 1937; Epstein, 1956*a* and 1957*a*) and their structure was uniform (Fig. 1 to 4) and identical with that of the Rous virus (Epstein, 1957*a*); such particles have never been observed in pellets made from either fluorocarbon-treated suspensions of vaccinia infected chick chorio-allantoic membranes (Epstein, 1958) or normal chorio-allantoic membranes (Holt and Epstein, 1958). More important by far, the results of the titration experiments (Table I) clearly show that when the suspensions were centrifuged their biological activity came to lie in the region of the pellets occupied by the particles (Experiments 1–4) and composed of almost nothing but particles (Fig. 2) in those cases where seven fluorocarbon treatments were used in the purification. Thus, the uniform formed particles can be identified as the Rous virus by virtue of the biological activity which they possessed.

This conclusion is re-inforced by the exactly comparable results obtained when vaccinia virus was treated in the same way as the Rous virus (Epstein, 1958). Since the work with the Rous and vaccinia viruses consisted of a series of identical experiments in which only the particle under test was varied, the results of each investigation support the validity of the other. It should be noticed, however, that when the biological activity of samples from the different regions of the Rous pellets was compared, that of the white zones containing the particles did not exceed that of the gelatinous areas quite as dramatically as was the case with the vaccinia pellets (Epstein, 1958). This is probably a consequence of the great difficulty involved in avoiding clumping and aggregation of the relatively small Rous virus when samples of the pellets were re-suspended for biological testing. On the other hand, the fact that the Rous virus needed more treatments with fluorocarbon than vaccinia in order to separate it from formed cell constituents, is perhaps due to differences in the fine structure of the cells infected with the two viruses. This may perhaps also explain the presence of the occasional cell fragments found associated with the Rous virus but not with vaccinia virus.

Besides allowing the viral nature of the particles associated with solid Rous tumours to be established, the present work has shown that these particles can be isolated, in a circumscribed area of pellet, almost entirely free from host cell elements (Fig. 2). This represents a considerable advance, for the pellets obtained from " purified " suspensions of the Rous virus which were sectioned and examined with the electron microscope by Bernhard, Oberling and Vigier (1956) consisted almost wholly of cell débris containing only extremely rare unidentified particles of the shape and size appropriate for the Rous virus. Even where this virus has been isolated by the method of Moloney (1956), pellets examined in the electron microscope were composed substantially of formed cell débris with groups of aggregated particles embedded in it (Haguenau, Moloney and Dalton, 1957).

The presence of the gelatinous material in the pellets shows that fluorocarbon treatment left the Rous virus associated with certain contaminating substances just as occurred in the case of vaccinia virus (Epstein, 1958). In both cases the substances seem to be of the same kind and include much free deoxyribonucleic acid (Holt and Epstein, 1958 ; Epstein and Holt, 1958). The latter contaminant in particular precludes the use of fluorocarbon-treated Rous virus preparations for chemical analysis of the virus for the same reasons as have been discussed with reference to vaccinia virus isolated in this way (Epstein, 1958). It seems probable, however, that the methods suggested for freeing fluorocarbon-treated vaccinia virus from the deoxyribonucleic acid contaminating it (Holt and Epstein, 1958) would apply equally well in the case of the Rous virus.

Even without adopting further purification measures, as work reported elsewhere shows (Epstein and Holt, 1958), the dense white zones of the pellets containing the Rous virus freed by the fluorocarbon from formed host contaminants (Fig. 2) afford an ideal source of purified material for the cytochemical study of the composition of the virus.

### SUMMARY

Experiments are described in which a fluorocarbon ($CF_2Cl$–$CCl_2F$) was used to prepare suspensions of the particles which occur in association with the cells of the Rous sarcoma. Nodules of solid Rous tumours were grown on the chorio-allantoic membranes of chick embryos and their virus-like particles were separated from host cell constituents at a water-fluorocarbon interface ; the suspensions were subjected to high speed centrifugation for one hour and the pellets obtained were fixed, embedded and sectioned for electron microscopy. Direct observation of the sectioned pellets with the electron microscope has been used as a check on their purity. In combination with the morphological work, tests for biological activity have been made on the material at each stage in the experiments in order to investigate the nature of the particles.

The results show that fluorocarbon treatment enabled suspensions of virus-like particles to be made which were almost entirely free of recognisable formed host cell constituents but which nevertheless contained other important host substances. This was demonstrated by the fact that the pellets made by high speed centrifugation of suspensions treated by seven applications of fluorocarbon consisted of a firm white zone composed of spherical uniform particles about 75 m$\mu$ in diameter, as well as a gelatinous region. The particles had a fine structure indistinguishable from that of the Rous virus. The viral nature of the particles has been established by the results of the biological tests ; these have shown that about 99·9 per cent of the infectivity of the suspensions was eliminated by the gravitational force applied and that this activity came to reside, after the centrifugation, in that part of each pellet in which the particles were the only constant feature or, where seven fluorocarbon treatments had been used, the only feature.

The author is most grateful to Dr. P. Armitage of the Medical Research Council's Statistical Research Unit (London School of Hygiene and Tropical Medicine) for his kind help with, and approval of, the presentation and evaluation of the results of the titrations.

The expenses of this investigation were borne by the British Empire Cancer Campaign.

### REFERENCES

Andrewes, C. H.—(1936) *J. Path. Bact.*, **43**, 23.
Benedetti, E. L. and Bernhard, W.—(1957) *C.R. Acad. Sci., Paris*, **244**, 2204.
Bernhard, W., Oberling, C. and Vigier, P.—(1956) *Bull. Ass. franç. Cancer*, **43**, 407.
Beveridge, W. I. B. and Burnet, F. M.—(1946) *Spec. Rep. Ser. med. Res. Coun. Lond.* (H.M. Stationery Office), No. 256, p. 15.
Caulfield, J. B.—(1957) *J. biophys. biochem. Cytol.*, **3**, 827.
Claude, A.—(1937) *J. exp. Med.*, **66**, 59.
Craigie, J.—(1949) *Brit. J. Cancer*, **3**, 249.
Elford, W. J. and Andrewes, C. H.—(1935) *Brit. J. exp. Path.*, **16**, 61.
Epstein, M. A.—(1951) *Ann. Rep. Brit. Emp. Cancer Campgn*, **29**, 59.—(1956*a*) *Brit. J. Cancer*, **10**, 33.—(1956*b*) *Ann. Rep. Brit. Emp. Cancer Campgn*, **34**, 89.—(1957*a*) *Brit. J. Cancer*, **11**, 268.—(1957*b*) *J. biophys. biochem. Cytol.*, **3**, 851.—(1958) *Brit. J. exp. Path.*, in press.
*Idem* and Holt, S. J.—(1958) *Brit. J. Cancer*, in press.
Gaylord, W. H.—(1955) *Cancer Res.*, **15**, 80.
Gessler, A. E., Bender, C. E. and Parkinson, M. C.—(1956) *Trans. N.Y. Acad. Sci.*, II, **18**, 701.
Haguenau, F., Moloney, J. B. and Dalton, A. J.—(1957) *C.R. Acad. Sci., Paris*, **245**, 2230.
Holt, S. J. and Epstein, M. A.—(1958) *Brit. J. exp. Path.*, in press.
Luft, J. H.—(1956) *J. biophys. biochem. Cytol.*, **2**, 779.
McIntosh, J. and Selbie, F. R.—(1937) *Brit. J. exp. Path.*, **18**, 162.
Moloney, J. B.—(1956) *J. nat. Cancer Inst.*, **16**, 877.
Palade, G. E.—(1952) *J. exp. Med.*, **95**, 285.
Prince, A. M.—(1958) *J. nat. Cancer Inst.*, **20**, 147.
Reed, L. G. and Muench, H.—(1938) *Amer. J. Hyg.*, **27**, 493.
Rouiller, C., Haguenau, F., Golde, A. and Lacour, F.—(1956) *Bull. Ass. franç. Cancer*, **43**, 10.